# Reflections on the Indications for Prostatectomy

**Published:** 1961-07

**Authors:** J. P. Mitchell

**Affiliations:** Urological Surgeon, United Bristol Hospitals


### REFLECTIONS ON THE INDICATIONS FOR PROSTATECTOMY

BY

J. P. MITCHELL

*Urological Surgeon, United Bristol Hospitals*

As recently as twenty five years ago prostatectomy was a dangerous operation which in the majority of cases was performed in two stages, and the total time spent in hospital was anything from 8 to 12 weeks. The patient almost invariably had to resign himself to a period of urinary leakage from a fistula and this could only be controlled by means of appliances such as the Hamilton-Irving box or large cumbersome absorbent dressings such as moss packs. In either case, the inevitable accompanying odour made the patient feel that he was an abominable social outcast until the fistula finally dried up. Furthermore, the patient had to be reasonably fit to survive the operation of prostatectomy. The mortality figures published for this period are not a true reflection of the severity of the operation, as these figures did not include those who died after the first stage, that is to say, the suprapubic cystostomy. Although in the last twenty five years the figures for prostatectomy do not appear to have improved, a very much larger proportion of the patients suffering from prostatic obstruction are now offered the benefits of prostatectomy, and this operation is performed in one stage with no preliminary filtering off of the weaker ones by the first-stage suprapubic cystostomy.

### INDICATIONS FOR OPERATION

Broadly speaking, there are two indications for prostatectomy. The first is some definite evidence of back-pressure either on the upper urinary tract producing hydronephrosis, or on the lower urinary tract resulting in acute or chronic retention, trabeculation of the bladder wall, or merely a very large residual urine, which in fact is the early stage of chronic retention. The second indication for prostatectomy is the inconvenience that the enlarged gland causes the patient. Occasionally a patient with benign prostatic hypertrophy will present with haematuria. This may occur only once or twice, or it may persist for several days. It is, of course, essential to exclude any other cause for the haematuria by intravenous pyelography and by cystoscopy, but haematuria from an enlarged prostate is not *per se* an indication for prostatectomy, and many of the patients will never have another bleed, or may go many months or years before any further urinary symptoms develop. In the Department of Urology in Bristol in the past ten years protatectomy has been performed on only four occasions for heavy haematuria, in the absence of other marked obstructive symptoms.

In those patients with symptoms of prostatism without any clinical or radiological evidence of back-pressure on the bladder or upper urinary tract, assessment of the inconvenience that the enlarged prostate is causing to the patient often presents some difficulty. One patient will maintain that his sleep is disturbed, and his broken nights are causing him distress when he only has to get up twice during the normal sleeping hours to pass water. On the other hand, some men will accept the fact that they have to wake up four or five times to pass water, but because they are able to get to sleep again immediately, they do not feel that this is sufficient to justify a major operation. Diurnal frequency can also be very variable in the inconvenience it causes to the patient. Here, however, it is usually a question of social or professional duties. The old gentleman who has to attend Board meetings or to sit on the Bench as a Magistrate may find it very embarrassing if he cannot hold his water for longer than two hours, whereas the man who has retired to a quiet life in his garden and his greenhouse probably is not aware that he passes his water every hour. Urinary incontinence is easy to assess, as this is almost invariably associated with some degree of chronic retention, the incontinence being a form of overflow. Hesitancy is a symptom which usually distresses the patient in the middle of the night when he is disturbed by the desire to pass water, and he stands for several minutes or even half-an-hour in the cold before he is able to produce a trickle of urine. Another man may be troubled by day, particularly in a public urinal when, for example, during the interval at the theatre he finds he is quite unable to produce any urine because others are present, and he has to return to his seat and endure the next act in great discomfort. Journeys by car or train can cause considerable embarassment to the urinary flow in patients who otherwise may have little trouble with micturition. This may be due to vascular congestion or possibly to vibration transmitted to the sacrum, producing reflex spasm of the bladder neck via the nervi erigentes.

The inconvenience which prostatic symptoms cause to the patient can only be assessed by the patient himself, and it is the doctor's duty to advise the patient firstly as to how much relief this operation can give, secondly, on the relatively low mortality in patients who are otherwise fit.

Patients may sometimes ask, even though their symptoms at the time are minimal, whether they ought not to undergo the operation in view of the progressive nature of the disease and the inevitable deterioration of their physical fitness as they grow older. This form of prophylactic prostatectomy can rarely be justified in the light of modern anaesthetic advances, where age alone adds comparatively little to the operative risks. Although prostatic hypertrophy is always a progressive condition, it is impossible to assess the rate at which progress will occur. This progress cannot be assessed on the rate of development of earlier symptoms as these minimal troubles can remain quite unchanged for several years; nor could a prognosis ever be given on the chances of the development of retention.

In two instances we may be forced to accept a lesser degree of symptoms as an indication for surgery. The first is that of the chronic bronchitic, who may be a very bad risk for an emergency prostatectomy in winter when his chest is at its worst, whereas the same operation for him in early summer could be a less hazardous procedure and would give him the rest of the summer months for his convalescence. The other instance where assessment is always difficult is that of the man who is going abroad for business or professional reasons or even for a prolonged holiday, perhaps to celebrate the beginning of his retirement. He is apprehensive at the thought of being rushed into hospital in a foreign country where he may not be fluent in the language, and he will be far from his wife and family. Admittedly air transport reduces this problem for the businessman whose fare is paid, and who can be despatched home at short notice; but the traveller who has retired on a pension, and has probably saved for many years for this journey to foreign parts perhaps to see one of his children who has emigrated, is faced with a serious dilemma. Here it is probably wise to accept symptoms of rather less severity and advise surgery so that he can make his journey with complete peace of mind.

*Fitness for operation*

Because of the age of most patients for whom prostatic surgery is contemplated, we find in the majority either some cardiac or respiratory complication, or both. A physical examination of the patient will give us little information as to whether that patient will stand an anaesthetic and surgery. The enquiries most helpful in assessing the physical fitness of the patient are those directed to an assessment of his mode of life during the past few months. Has he been up and about and leading a reasonably active life? Has he a garden that he looks after, or does he do the shopping for his wife?

Finally, if he can get out of bed, walk round it, and get back in again without assistance, he is almost certainly fit to stand a prostate operation.

*Contra-indications to operation*

The contra-indications to prostatic surgery are either advanced cerebral disease, or a patient bedridden because of cardiac, respiratory or locomotor failure, or a combination of these. Such bedridden patients will probably be just as well off with an indwelling catheter, and certainly will be much easier to manage. The post-operative results if surgery is attempted in a bedridden patient are never so successful, presumably because these patients tend to develop a sump of infected urine in the base of the bladder and pass only the clear supernatent fluid, which results in a severe basal cystitis. Even though the residual urine may be only 1 or 2 ounces, this remains in the *bas fond* of the bladder stagnating into thick pus.

### FURTHER CONSIDERATIONS

*The size of the prostate*

Examination of the prostate per rectum can give little help in assessing the need for surgery, as the size of the prostate bears little relationship to the degree of obstruction it may be causing. Obstruction results from the prostate encroaching on the urethral lumen and from interference with the normal musculature of the urethral wall. Around a prostatic adenoma the normal prostatic tissue is compressed into a capsule, the so-called false capsule of the prostate, and this tissue may consist of a considerable amount of fibrous stroma. If this prostatic capsule is thick and rigid, then only a small amount of enlargement of the prostate will give rise to symptoms, whereas if the capsule is lax, and distends easily with enlargement of the adenoma, then a prostate can attain a considerable size before any symptoms develop at all.

The small prostate which amounts to little more than hypertrophy of the bladder neck is likely to present with a gradual increase in frequency and progressive development of back-pressure in the form of chronic retention, whereas with a larger prostate the patient will complain of obstruction to the flow or ultimately acute retention. Therefore the size of the prostate can give some indication of the type of symptoms to expect in the future, though it cannot indicate how soon the patient's condition is likely to require surgical intervention.

Cystoscopy can give a crude estimate of the degree of back-pressure on the bladder by revealing the extent of the muscular trabeculation that has occured on the bladder wall, with possibly in addition the formation of saccules or even a diverticulum. Where there has been haematuria as a presenting symptom, cystoscopy has to be performed to exclude any neoplasm of the bladder wall. Unfortunately, cystoscopy introduces the risk of throwing the patient into retention shortly afterwards, but it is fair to say that a prostate which produces acute retention immediately after cystoscopy has been performed would, in all probability, have caused obstruction in the near future, even in the absence of the cystoscopy.

*Indications for transurethral resection as opposed to open surgery*

In assessing the risk of an operation on the prostate gland, it is a fallacy to imagine that a transurethral resection is any less in severity than an open prostatectomy by either the transvesical or retropubic route. Transurethral resection has certain advantages in a cardiac or bronchitic patient in that the operation can be done in two stages, or can be stopped at short notice if the anaesthetist is anxious. In the post-operative stage a bronchitic patient can cough without the discomfort of an abdominal wound. On the other hand, the systemic disturbance to the patient and the risk of haemorrhage is no less from the transurethral operation.

*Age of patients*

One could almost say that no patient is too old for prostatectomy. On the other hand, the older the patient the higher is the incidence of some other complicating factor such as cerebral, cardiac, or respiratory disease. The most helpful approach in assessing an aged patient who is a prospective candidate for prostatectomy is to consider his natural expectation of life. From the Registrar General's figures, a patient who has reached the age of 70 has an expectation of nine years; a patient who has reached the age of 75 can expect to live a further seven years. To interpret these figures more vividly, they could be interpreted as saying that 50 per cent of 75-year-olds are going to survive to a greater age than 82. Although most surgeons can quote occasional successes of prostatic surgery amongst nonogenarians, we have to admit that a proportion of the latter are leading only a vegetative life, and it is possible that they would have been just as well off with a permanent indwelling catheter.

*The alternative to prostatectomy*

In the event of prostatectomy being contra-indicated, we are left with only two alternatives in a patient who already has retention, namely, a permanent indwelling catheter or a permanent suprapubic cystostomy. A few patients will tolerate a suprapubic cystostomy with complete equanimity but to the majority of patients it is irksome and even painful. A permanent indwelling urethral catheter on the other hand gives comparatively little discomfort. A Foley catheter has to be used, this being the only self-retaining form of urethral catheter, but these catheters have a very narrow lumen for drainage of the bladder, as a large part of their lumen is taken up by the tube which inflates the self-retaining balloon. Unless the catheter is fairly large (22 Charriere or more) there is likely to be considerable trouble with blockage of the catheter from débris and phosphatic incrustations. These catheters should always be changed at 3 or 4-week intervals, depending on the speed with which the patient forms phosphatic plaques on the catheter. Unless patients are bedridden they will usually prefer to take the risk of surgery rather than put up with the discomfort and inconvenience of any form of catheter.

*Emergency prostatectomy*

In the past there has been much discussion about the optimum time for surgery in urinary retention, both acute and chronic. Wilson Hey was the first and foremost exponent of immediate surgery for acute retention of urine in his efforts to reduce the incidence and severity of post-operative infections. He decried the use of catheters in the relief of acute retention, and would operate without preliminary cystoscopy in order to avoid passing any instrument, thereby hoping to reduce the risk of organisms being carried into the bladder from the external urinary meatus. Indeed, even the drainage catheter placed in the urethra at the end of the operation was passed down the urethra fron the bladder rather than in the customary direction from the external meatus. Certainly this routine contributed much towards reducing the severity of post-prostatectomy infection and its complications, and Wilson Hey's enthusiasm focussed our attention on the possible causes of these infections.

It is often impossible by day to arrange for an operating theatre at short notice without inconveniencing colleagues, At night, after the full use of the theatres all day, there might be a queue of emergencies waiting in the wards, and "immediate prostatectomy" would have to take its turn. The result was that acute retention came to be regarded as an emergency only in that it had to have surgery within 48 hours of the passage of the urethral catheter. One further disadvantage of immediate surgery was the possibility that the case would prove at cystoscopy to be more suitable for a transurethral resection, an operation which is difficult to perform as an emergency because the night theatre staff are rarely familiar with the procedure or the instrument.

### PRELIMINARY DRAINAGE IN CHRONIC URINARY RETENTION

An equally controversial question is how and when to proceed in cases of chronic urinary retention. Should the bladder be given a period of preliminary drainage in the hope of improving renal function and resting the atonic bladder? If the urine was already heavily infected there was little to lose by preliminary drainage, and renal function was sure to improve with release of the urinary back-pressure. The problem was more complex in the case of the chronic retention with sterile urine and hydronephrosis, when the blood urea might be considerably raised. This, in fact, is one of the worst surgical risks in urology, as until recent years the condition of the patient was bound to deteriorate whatever was done. If the bladder was only drained by an indwelling catheter (urethral, perineal or suprapubic) then infection occurred, and inevitably the damaged kidneys which had already suffered from back-pressure now had to face infection, the effects of which outweighed the relief of the back-pressure. For this reason many urologists were in favour of immediate surgery in chronic urinary retention, believing that they were then operating on the patient before his condition deteriorated from infection. It was considered that careful correction of electrolytes and fluid balance gave the patient a good chance of recovery from the immediate post-operative stage, when the blood loss was at its worst; and he could then face the infective complications knowing the operation was over, and with the advantage of an unobstructed urethra which could be used normally as soon as the bladder tone recovered. Certainly it seemed that those who were subjected to the major surgery of prostatectomy did no worse than the others in whom simple catheter drainage had been instituted, and these latter still had to face their operation.

In Bristol until recently early surgery has been preferred for chronic urinary retention when the urine was clear and sterile. In the light of previous investigations into urinary infections (Miller *et al.*, 1958, 1960 a, b), and the use of the long polythene catheter of very narrow calibre (Gibbon, 1958), our views on the time for surgical intervention are now being modified. Although there is so much in favour of early surgery, it is impossible to deny that disadvantages exist.

The major anxiety with immediate surgery is the problem of increased haemorrhage due to congestion of the decompressed bladder wall, which becomes engorged with dilated blood vessels. The risk of haemorrhage is also increased by infection ascending via the catheter and thence up to the kidneys, producing an acute haemorrhagic pyelonephritis superimposed on kidneys already damaged by back-pressure. There is a tendency for uraemic patients to bleed, and at the same time uraemia depresses the haemopoietic function of the bone marrow and causes further delay in the recovery of the haemoglobin level. A large catheter (plastic Harris size 24 Charriere) has to be used in the immediate post-operative stage, and there is little doubt that the severity of the urethral reaction it produces is directly proportional to the size of the catheter.

Now that infection is no longer accepted as an inevitable sequel to an indwelling catheter, and the size of the catheters has been reduced, there is a move back to preliminary drainage by an indwelling urethral catheter inserted and maintained with full sterile precautions and connected to a closed drainage bottle. The Gibbon catheter, which is basically a 4-foot length of fine polythene tubing, varies from 6 to 12 Charriere and has a lumen wide enough to drain clear and uninfected urine, though it will not allow blood clots or infected débris to pass.

The bladder distended with gross chronic retention will have an atonic musculature. A period of drainage will therefore be needed before the muscle of this bladder wall will recover its tone.

The treatment of chronic bladder retention can be summarised under the following headings: patients with or without renal failure, and those with or without urinary infection. There is never any doubt when infection has occurred in these patients as the retained urine ensures that the organisms thrive and the bladder contains offensive cloudy urine, sometimes as thick as pea soup.

(*a*) *Chronic retention with infected urine*

Preliminary drainage allows an opportunity of treating the infection irrespective of renal damage. If the blood urea is raised then the drainage will relieve the back-pressure effects on the kidneys.

(*b*) *Chronic retention with sterile urine*

If the blood urea is normal, no advantage can be gained from preliminary drainage and routine definitive surgery can be performed without any previous instrumentation of the urethra.

If the blood urea is raised, then provided full precautions are taken against the introduction of infection, the preliminary catheter drainage will improve the renal function, and operation can be delayed until the patient is no longer uraemic. The serious risk of introducing infection cannot be overstressed, as these patients so readily succumb to an ascending pyelonephritis. It is in these cases that the catheter can be lethal, and should only be introduced with full sterile technique and maintained on a ward where the nursing staff are fully trained in the care of a closed drainage apparatus.

*The senile patient with chronic retention*

Occasionally the problem of an elderly patient with chronic retention and minimal urinary symptoms has to be faced. He may have respiratory, cardiac, cerebral, or other complications, which by their severity contra-indicate surgery. In these patients the question arises whether to insert an indwelling catheter or not, despite the chronic retention. If there is infection in the urine, then no choice remains but to catheterise. On the other hand, in patients with sterile urine a case can be made for withholding the catheter. These patients can sometimes continue for several months without obvious increase in the size of the bladder or any rise in the blood urea level, but a regular check on both these factors must be kept. In some of these elderly patients the residual urine can be variable, depending on their activity, When they have been tided over an attack of congestive heart failure or bronchitis, the residual urine may decrease as the patient begins to get about.

### ADVICE TO PATIENTS NOT NEEDING IMMEDIATE SURGICAL TREATMENT

Finally, what advice should be given to the patient with minimal symptoms, who wishes to avoid aggravating his frequency or precipitating acute urinary retention? So often a patient coming into hospital for prostatectomy will have been restricting his fluids to reduce his output even to the extent of inducing a state of dehydration, as can be seen by gently lifting his skin between the fingers. It is very doubtful whether excessive restriction of fluids gives these patients any relief from their frequency. This may sound paradoxical, but if they are encouraged to drink a little more, say 2 pints a day, they find their frequency remains the same. It is possible that this is because a less concentrated urine provokes less irritation of the bladder mucosa.

Acute urinary retention is often precipitated by allowing over-distention of the bladder. This may be due to the social inconvenience of emptying the bladder, or to a lulling of the sense of urgency to pass water as a result of a quiet evening on some form of alcohol. Once the bladder distends, the normal process of initiating micturition is impaired, and it quickly goes into complete retention. It seems unlikely that alcohol itself can have any direct effect on the bladder-neck sphincters or the prostate, but the imbibing of alcohol is often associated with some convivial occasion and the urge to pass water may be disregarded until it is too late.

The advice then to a patient with early prostatic symptoms should be to drink approximately 2 pints of fluid per day, and never to disobey the call to pass water. He should not hold his water longer than 2–3 hours, and when making a train or car journey he should deliberately relieve himself immediately beforehand. If possible, he should avoid public urinals and try to retire to the privacy of a single toilet where nervous agitation will not disturb his urethral sphincters. Restriction of fluid just before bedtime can reduce nocturnal frequency, as concentrated urine does not seem to produce frequency so readily once the patient is asleep. Finally, there is the vexed question of alcohol; one need only advise moderation, not only in alcohol but in actual volume of fluid. To condemn these patients to an abstemious life may drive some into surgery, as they may feel that life without a prostate is infinitely preferable to life without a glass of ale.

Oestrogens have been tried in the past, but they are of no benefit in benign prostatic hypertrophy. Their use should be reserved for the malignant gland.

*Combined surgery*

The patient who has decided to have an operation for relief of his prostatic obstruction may ask for "another small job to be done at the same time", and the commonest request is for repair of a hernia. Although this may seem perfectly feasible, and should not increase the risk or the time of operation to any great extent, it does allow a greater chance of wound infection and breakdown—a transient discharge from the prostatectomy wound could infect the hernia repair, and *vice versa*. The surgeon is therefore usually reluctant to combine prostatectomy with any other procedure.

REFERENCES

Miller A., Gillespie W. A., Linton K. B., Slade N. and Mitchell J. P. (1958). *Lancet,* **ii,** 608.
Miller A., Gillespie W. A., Linton K. B., Slade N. and Mitchell J. P. (1960a). *Lancet* **i,** 310.
Miller A., Gillespie W. A., Linton K. B., Slade N. and Mitchell J. P. (1960b). *Lancet* **ii,** 886.
Gibbon N. (1958) *Brit. J. Urol.,* **30,** 1.

